# Temporal dynamics of floral characteristics and reproduction output of *Impatiens oxyanthera* under warming

**DOI:** 10.1093/aobpla/plaf046

**Published:** 2025-08-30

**Authors:** Qiao Yan, Qiuhai Su, Dengfei Li, Qiong Wang

**Affiliations:** Key Laboratory of Southwest China Wildlife Resource Conservation (Ministry of Education), China West Normal University, No.1 Shida Road, Nanchong, Sichuan 637009, China; Key Laboratory of Southwest China Wildlife Resource Conservation (Ministry of Education), China West Normal University, No.1 Shida Road, Nanchong, Sichuan 637009, China; Key Laboratory of Southwest China Wildlife Resource Conservation (Ministry of Education), China West Normal University, No.1 Shida Road, Nanchong, Sichuan 637009, China; Key Laboratory of Southwest China Wildlife Resource Conservation (Ministry of Education), China West Normal University, No.1 Shida Road, Nanchong, Sichuan 637009, China; Populations & Communities

**Keywords:** climate warming, *Impatiens oxyanthera*, flower traits, flowering period, reproductive success

## Abstract

Climate warming threatens plant sexual reproduction, and plants with extended flowering can experience distinct biotic and abiotic environments across the season. Therefore, responses and adaptations of plant reproduction to warming may vary across the season. Our aim was to examine how climate warming affects plant floral traits and reproductive success across different phenological stages within a single flowering season. In this study, infrared heaters were used to simulate warming (+1.5°C) during the growing season of *Impatiens oxyanthera*. Flowering was divided into early, middle, and late time-periods based on the flowering onset and end dates of the experimental population. The changes in floral and reproductive characteristics, as well as their relationships across these three time periods, were investigated under warming conditions. Our study on *I. oxyanthera* demonstrates that warming significantly delayed flowering onset, reduced the number of flowers per plant, and decreased both the length and curvature of nectar spurs. Warming also disrupted correlations between floral traits to some extent compared with the control. Flowers that opened during the late period were smaller, had fewer ovules but more nectar, and produced fewer filled seeds. Warming exerted period-specific impacts on nectar spur length, reducing it during the late flowering period compared with the control treatment but not during the early or middle periods. However, the changes in floral traits caused by the interaction of warming and flowering period did not significantly affect reproductive success at the single-fruit level. These findings highlight the temporal heterogeneity of plant responses to climate warming and suggest that potential buffering mechanisms might contribute to maintaining reproductive outcomes under moderate warming conditions.

## Teaser

This study investigates how climate warming affects floral traits and reproductive success in Impatiens oxyanthera across different flowering periods. Using infrared heaters in a field experiment, the research reveals that warming delays flowering onset, alters floral characteristics, and affects plant-pollinator interactions, but does not significantly reduce fruit or seed production. The findings highlight the complex, stage-dependent ecological responses of plants to climate change.

## Introduction

Floral traits play a pivotal role in shaping plant reproductive success by regulating resource allocation, mediating interactions with pollinators, and influencing mating opportunities. Flowering phenology, which reflects the temporal characteristics of plant flowering (e.g. flowering onset, peak time), is a core parameter for studying the temporal dynamics of pollination (CaraDonna et al. 2014). Specifically, earlier flowering onset and a longer flowering period favour increased flower production (Inouye 2008, Dorji et al. 2020), thereby enhancing the potential for reproductive output. Floral traits such as size, shape, colour, rewards, and scent profiles act as critical signals or food sources to attract pollinators, thereby facilitating efficient pollen transfer and plant reproductive success (Galen 1999, Parachnowitsch and Kessler 2010). Larger floral displays enhance pollinator attraction through increased visual conspicuity and greater advertisement of nectar/pollen rewards (Parachnowitsch and Kessler 2010, Amasino et al. 2017). Furthermore, corolla tube size and nectar spur morphology determine pollinator accessibility and mechanical fit, while specialized structures may act as filters for specific pollinators (Young 2008, Trunschke et al. 2020). Nectar is the most important reward that plants supply for pollinators. Nectar production and sugar composition modulate pollinator foraging behaviour, with higher nectar rewards correlating with prolonged visitation and enhanced pollen deposition and removal efficiency (Mitchell et al. 2009). Therefore, floral traits exert an influence on plant sexual reproduction via their effects on resource acquisition and pollen availability.

The Sixth Assessment Report of the IPCC predicted that the global average temperature would exceed 1.5°C above pre-industrial levels within the next 20 years due to greenhouse gas accumulation (IPCC 2021). Elevated temperatures profoundly influence the expression of floral traits through direct physiological stress and altered resource allocation, with cascading effects on plant-pollinator interactions and reproductive success. For early flowering species, warming usually advances flowering onset and extends flowering duration (Dorji et al. 2020, Descamps et al. 2021). However, in late-flowering species, warming tends to delay flowering onset (Sherry et al. 2007). Warming-induced flowering phenology changes potentially disrupt synchrony between plants and pollinators unless pollinators can keep up with plants’ pace. Concurrently, warming reduces the size and number of flowers through decreasing resource allocation, restricting flower development (Hoover et al. 2012, Descamps et al. 2021). Warming-caused narrower corolla tubes or reduced spur lengths may lead to an increase in size mismatch between plants and pollinators and thus a reduction in plant reproductive success (Fenster et al. 2004, Tao et al. 2024). At the individual flower level, warming decreases nectar volume resulting from water deficit and alters sugar composition (Takkis et al. 2018, Kuppler and Kotowska 2021). These changes in floral traits can alter pollinator visitation patterns, reduce pollen transfer efficiency (Mitchell et al. 2009) and thus decrease fruit production (Zi et al. 2023). Increased temperatures may prompt plants to adjust their reproductive strategies, such as altering the trade-off between seed quality and quantity, to adapt to environmental changes, but these effects vary depending on factors such as plant species, ecology, and experimental conditions (Kuppler and Kotowska 2021). Collectively, these responses underscore how warming reshapes plant fitness via both direct physiological effects and indirect ecological feedback.

Plants face different biological (e.g. pollinators, herbivores) and non-biological (e.g. light, temperature, water) resources and environments at different flowering periods and thus their reproductive outputs and strategies may vary over time (Sisodia et al. 2020, Lee et al. 2023). For example, *Primula sikkimensis* produces earlier compared with later in the flowering season, which results in exposure to fewer pollinator species, reduced visitation frequency, and consequently lower female fitness (Cha et al. 2023). When floral competition is low, early-opening flowers tend to allocate higher nectar volumes to attract pollinators, whereas later-opening flowers may reduce nectar investment due to resource depletion or prioritized allocation to seed maturation (Parachnowitsch and Kessler 2010, Russell and McFrederick 2022, Benadi et al. 2023). Many plants exhibit higher ovule numbers in early season flowers, particularly under conditions of abundant resources and active pollinator visitation (Obeso 2002), a strategy that ensures reproductive success (Knight et al. 2005). Later, in the flowering season, plants may face depletion of photosynthetic products or mineral nutrients, leading to insufficient resources for ovule development and increased seed abortion rates (Zinn et al. 2010). During the flowering season, there can be differences in the types, quantities, visiting behaviours, and pollination efficiency of pollinators. For example, plants at their peak flowering have a higher flower density, which is more attractive to pollinators and increase visitation frequency, thus improving the reproduction success rate (Weber and Kolb 2013, Ruane et al. 2014). Previous studies have primarily focused on comparing the effects of warming on reproductive investment between early-flowering and late-flowering individuals (Sherry et al. 2007, Zhang et al. 2024) but have not examined the differences in these effects among distinct stages during flowering.

Here, we simulated warming with infrared heaters during the growing season of *Impatiens oxyanthera* and compared the differences in the effects of warming on the floral traits and plant reproductive success among early, middle, and late flowering periods. Our study aimed to address two key questions: (i) How does warming affect floral traits during different flowering periods? (ii) Are changes in floral traits during different flowering periods more or less likely to affect reproductive output under warming?

## Materials and methods

### Study site

The study was conducted in Mount Emei, China (29°36′16″ N, 103°22′02″ E, 932 m a.s.l., Supplementary Figure S1), situated on the eastern Tibetan Plateau (Gu and Li 2008). This region has been identified as among the globally most sensitive mountains to climate warming (Liu et al. 2021). Over the past 30 years (1991–2019), air temperatures at the top and the foot of Mount Emei have increased by 0.75 and 1.35°C, respectively (Appendix S2: Supplementary Figure S2). Mount Emei below 1500 m a.s.l. has a subtropical humid monsoon climate, with abundant rainfall from May to September (Tang 2006). The annual average temperature is 10–17°C, and the annual average rainfall is 1593–1990 mm (Liu 1992). Mount Emei is the concentrated distribution and differentiation area of *Impatiens* spp. in China (Xiang 2011), including 23 wild *Impatiens* spp. and 12 *Impatiens* spp. endemic to Mount Emei (Li and Shi 2007).

### Plant materials

The genus *Impatiens* have a rich variety of species, with more than 1000 species worldwide (POWO 2020). *Impatiens* spp. has important ornamental and medicinal values (Li et al. 2021a, Ashagrie et al. 2023) and is also an important component of understory vegetation (Chen 2001). *Impatiens* spp. are extremely sensitive to their growing environment and prefer a humid and cool environment (Chen 2001), thus being sensitive to temperature changes (Wang et al. 2013, Descamps et al. 2021, Tao et al. 2024). Warming delays flowering phenology, reduces the number or size of flowers, and decreases reproductive output in *Impatiens* plants (Wang et al. 2013, Descamps et al. 2021). *Impatiens* spp. usually have a long flowering period, blooming from July to September (Yu 2012).

*Impatiens oxyanthera* is a perennial herb endemic to China. This species has red or lilac flowers with some red stripes on funnel-shaped labellum, and has a long and curved nectar spur at the base of the labellum. The flowers are hermaphrodite, and their lifespan is 5–7 days, with a male phase of 3–4 days and a female phase of 2–3 days. It has a wide distribution range, occurring at altitudes from 500 to 2500 m above sea level (Yu 2012, Wang et al. 2013). *Impatiens oxyanthera* was selected as the experimental material for the following reasons: (i) *I. oxyanthera* has a long flowering period (from August to November), large flowers, a facultative outcrossing breeding system and requires pollinators, with its main pollinator being *Bombus trifasciatus*. (ii) The floral characteristics of *I. oxyanthera* are sensitive to warming (Wang et al. 2013, Tao et al. 2024), and the population of wild *I. oxyanthera* significantly declined from 2010 to 2025 (personal long-term observation). (iii) *I. oxyanthera* has high ornamental value (Wang 2008).

### Experimental design

Wild seedlings of *I. oxyanthera* were randomly collected within 1 km of the experimental site, which is located in open, flat agricultural land. On 13 April 2023, 360 wild seedlings of *I. oxyanthera*, each 9–11 cm in height and with similar growth conditions, were selected near the experimental plots. The seedlings were transplanted with their native soil into pots measuring 30 cm in diameter and 25 cm in height. The soil in the pots was loosened and finely crumbled to ensure optimal growing conditions. All plants were transplanted with soil (one plant in each pot) and randomly assigned to 12 experimental plots, divided into two rows with a 1-metre interval between each plot (Supplementary Figure S3). Once all seedlings had survived, the infrared heaters were turned on for the warming treatment on 24 May 2023. Six of these plots were randomly selected for elevated temperatures using six infrared heaters (Kalglo Electronics Inc, Bethlehem, PA, USA), and the remaining six plots served as the control (Supplementary Figures S3 and S4). Each infrared heater has a power of 2 KW and a bottom area of 165 cm × 15 cm and was suspended at a height of 1.7 m from the top of the flower pot in the middle of the plot. Six wooden boards with the same bottom area were suspended at the same location and height in the control plots to simulate the shading effects of the infrared heaters (Sherry et al. 2007). These warmed plots were heated for 24 h every day. Every 10 days, the infrared heaters in the warmed plots and the wooden boards in the control plots were rotated 45 degrees clockwise. During the warming period, excluding the days when the infrared heaters were turned off, the average air and soil temperatures in the warmed plots were 1.524°C and 1.840°C higher, respectively, than those in the control plots. Meanwhile, the air humidity in the control plots was 2.798% higher than that in the warmed plots throughout the growing season. The magnitude of simulated warming aligns with both the IPCC’s prediction of a 1.5°C increase in global average surface temperature above pre-industrial levels by the end of the 21st century (IPCC 2021) and the actual warming observed at the study site over the past 30 years. Because *I. oxyanthera* prefers a shady and humid environment, a shade net with a transmittance of (26.83 ± 0.66)% was covered above the experimental plots to simulate the light environment of natural habitats. In September, 2023, 56 of 180 warmed plants died, and 124 plants survived, of which 2 plants died during the experiment. Among the 180 control plants, 14 plants died and 166 plants survived. The data of subsequent experiments were based on these 288 plants.

The infrared heaters were turned off from 19 July 2023 to 5 September 2023 when air temperature often exceeded 30°C in the control conditions. On 13 October 2023, the heaters were operated alternately, with three heaters running daily for 24-h rotation due to insufficient power supply. The simulated warming was stopped on 20 October 2023. To ensure an adequate water supply during the experiment, an automatic sprinkler irrigation system (NADSTER, China) was installed at the experimental site. Plants were irrigated for 10 min (in spring and autumn) or 20 min (in summer) at 7 a.m. and 7 p.m. every day. Occasionally, manual supplementary watering was carried out in summer.

### Measurements of flower traits and sexual reproduction

Flowering started on 17 August 2023 and ended on 22 November 2023 for the whole experimental population. Accordingly, the flowering was divided into three time-periods: early (August 17—September 18), middle (September 19—October 20), and late (October 21—November 22). This division allowed for standardized comparisons across time and helped capture potential differences in plant responses to warming and environmental conditions throughout the flowering season. The flowering onset and end dates of all plants were recorded, and the duration of flowering was calculated. The number of flowers per plant was counted every 6 days because the lifespan of a single flower was 5–7 days (Wang et al. 2013). We calculated the proportion of plants with flowering onset or end out of total number of living plants in per plot to compare the differences in flowering onset and end time under the two warming treatments during different flowering periods. We also calculated the number of flowers of plants under the two treatments during different flowering periods.

**Figure 1.**
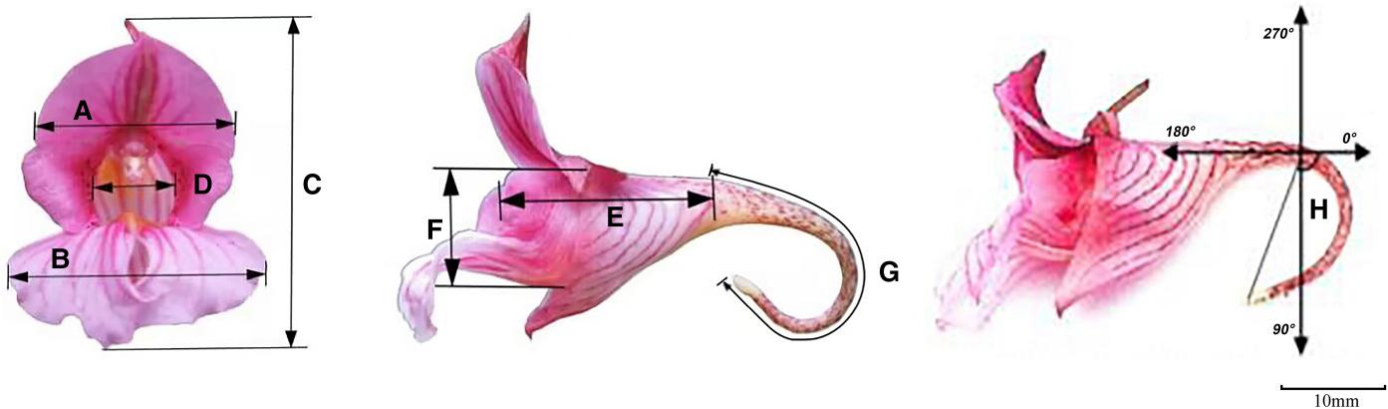
Determination of flower characteristics. (a) Vexillum width, (b) alae width, (c) corolla diameter, (d) channel width, (e) channel depth, (f) channel height, (g) nectar spur length, and (h) Nectar spur curvature.

The flower size (vexillum width, alae width, and corolla diameter), corolla tube size (channel depth, channel width, and channel height), nectar volume, nectar spur shape (nectar spur length, nectar spur curvature), and number of ovules of flowers were randomly measured for flowers on their first day of opening under both control and warming treatments across three flowering periods. Based on long-term personal observations, flower size remained stable throughout the lifespan of single flowers; therefore, only male-phase flowers on their first day of opening were measured. The flower size and corolla tube size were measured with a digital vernier caliper (CD-15AX, accuracy: 0.01 mm, Mitutoyo Corporation, Japan), and the nectar curvature was measured with a circular protractor (Fig. 1). The vexillum width is the distance of the widest part of vexillum, the alae width is the total width of the distal lobes of the alae, and the corolla diameter is the vertical distance from the highest point of the vexillum to the lowest point of the distal lobes of the alae. The corolla tube length was defined as the length of the basal lobe of the alae plus the depth of the labellum. The corolla tube width was measured as the horizontal distance between the petals forming the corolla opening. The corolla tube height was the vertical distance from the base of the vexillum to the base of the basal lobe of the alae. The nectar spur length was the distance from the end of the corolla tube to the tip of the nectar spur. The nectar spur curvature is the degree of curvature of nectar spur. The flowers used for measuring nectar volume were bagged from the mature flower bud stage to the morning on the first day of flowering to avoid being visited by pollinators. The nectar volume per flower was determined using a 10 μl flat-tip liquid-phase micro sampler (HAMILTON, Hamilton Company, Switzerland). The number of ovules per flower was determined by dissecting ovaries under an optical microscope (OLYMPUS CX23, Olympus Corporation, Japan). Please refer to the supporting information for the detailed number of repetitions (Supplementary Table S1).

Plant reproductive success was evaluated using key metrics including the number of filled seeds, unfilled seeds, unfertilized ovules, and seed set rate. It takes about a month from the withering of flowers to the maturity of fruits. Due to the low temperatures and early snowfall at the field site during the late time-period, flowers that bloomed during this phase did not develop into mature fruits or seeds. As a result, fruits from late-flowering plants were not included in the reproductive success analysis. The collected mature fruits were divided into two time-periods, the mid-period (September 19 to October 20) and the late-period (October 21 to November 22). Therefore, the reproductive characteristics of plants blooming during early and middle period were measured during middle and late periods, respectively. We counted the number of filled and unfilled seeds, as well as unfertilized ovules (retained on the fruit’s central axis) per fruit collected daily. The seed set rate was calculated by dividing the sum of filled and unfilled seeds by the total number of filled seeds, unfilled seeds, and unfertilized ovules. Please refer to the supporting information for the detailed number of repetitions (Supplementary Table S3).

### Monitoring of environmental factors

At the experimental site, four warmed plots and three control plots were randomly chosen. A temperature and humidity recorder (DS1923G, Maxim/Dallas Semiconductor Inc, USA) and a temperature sensor (DS1921G-F5, Maxim/Dallas Semiconductor Inc, USA) were positioned at the location of the first flower pot in the third row of each plot (Supplementary Figure S4). The temperature and humidity recorder, along with the temperature sensor, automatically recorded data once every hour to measure soil temperature, air temperature, and humidity. The temperature and humidity recorders were installed at a height of 20 cm above the flower pots to monitor the air temperature and humidity. To safeguard them from rainwater damage, a non-transparent wide-mouthed cup was placed 5 cm above the temperature and humidity recorder. After wrapping the temperature sensor with plastic film, it was inserted into a 10 cm soil depth beside the temperature and humidity metre within a flower pot to monitor the soil temperature. Since the temperature and humidity metres were installed at the plot edges, the actual temperatures of the warmed plots were higher than the measured values.

### Data analysis

All statistical analyses were carried out using the software R version 4.3.2 (R Core Team 2023). If the residuals of linear model met the normality and the variances were homogeneous, the data were fitted using linear models (LMs) or linear mixed effects models (LMMs). Otherwise, they were fitted using generalized linear models (GLMs, absence of random effect) or generalized linear mixed effects models (GLMMs, including random effect). Firstly, full factors LMMs and GLMMs were fitted using the measured flower traits (FT) and plant fitness (PF) as the responses, warming treatment and flowering period as predictors, and flower identity (Flower_ ID) or fruit identity (Fruit_ID) nested within individual plant identity (Plant_ID) nested within plot identity (Plot_ID) as random effects. Model: FT or PF ∼ warming treatment * flowering period + (1| Plot_ID/Plant_ ID/Flower_ID or Fruit_ID). If there was a deformed fit, the factors of random effects were modified until a normal fit appears. Family parameter was selected according to data types in GLMs and GLMMs. For count variables, models were fitted with a Poisson distribution. For ratio variables (e.g. seed set), models were fitted with a binomial distribution and logit link. For non-normally distributed continuous variables, we used models with a Gamma distribution. Residual normality was assessed via the *nortest* (Gross and Ligges 2015) package in R. All models utilized the *lme4* (Bates et al. 2019) and *Car* (Fox and Weisberg 2011) packages for fitting and Type III Wald χ^2^ tests, respectively. Post hoc Tukey-adjusted contrasts were computed via *emmeans* function (Lenth et al. 2020).

Flowering phenology traits, including initial flowering time (IFT) and end flowering time (EFT), were analysed using Accelerated Failure Time (AFT) models, a statistical framework in survival analysis that assesses the relationship between the time-to-event outcomes and explanatory variables. The analyses were conducted using the survreg function from the *survival* package in R (Therneau 2023). IFT and EFT were treated separately as time-to-event variables, with status indicating whether the flowering event was observed (1) or censored (0). Treatment was included as a categorical predictor, and a log-logistic distribution was specified to fit the survival curves. Model outputs were summarized, and ANOVA tests were performed to evaluate the significance of treatment effects. To investigate the effect of treatment (warming vs. control) on flowering duration and total number of flowers per plant, we fitted a GLMM with a poisson distribution and log link. We used the fixed effect of treatment and the random effect of Plant_ID nested in Plot_ID. For the proportion of flowering onset/end time per plot, binomial GLMs (logit link) was fitted to test warming treatment effects across early, middle, and late flowering periods, while plot-level flower counts were analysed using Gamma GLMs. Flower counts recorded every 6 days per plant throughout the entire flowering period were modelled using a zero-inflated Poisson GLMM (*glmmTMB* package). The model included fixed effects of experimental treatments and nested random effects of plot and plant (plot > plant) in both count component and zero-inflation component. Model diagnostics comprised checks for overdispersion and likelihood ratio tests, with Wald Z-tests (α = 0.05; Brooks et al. 2017) used to evaluate fixed effects.

Floral morphology (flower size, corolla tube size, nectar spur shape) and nectar volume were assessed using Gamma GLMs/GLMMs, with warming treatment, flowering period, and their interaction as fixed effects. Nectar volume models included Plant_ID as a random effect to accommodate repeated measures. Reproductive traits (seed set, number of filled seeds, number of ovules per flower) were analysed via three distinct models: (1) binomial GLMM for seed setting (nested random effects: Plot_ID/Plant_ID/Fruit_ID), (2) poisson GLMM for the number of filled seeds/unfertilized_ovules (random Plant_ID), (3) poisson GLM for the number of ovules per flower and (4) poisson GLMM for the number of mature fruits/seeds per plant (nested random effects: random Plot_ID/Plant_ID).

Pairwise Pearson correlations quantified associations between floral and reproductive traits within warming treatments during different flowering periods. Trait correlations were visualized using *corrplot* (Wei and Simko 2021) and *Hmisc* (Harrell 2024). To test whether the correlation coefficients between floral traits differed significantly between groups, we performed bootstrap correlation comparisons. The analysis was conducted in R using the *boot* packages (Canty and Ripley 2022). Linear regressions were used to test relationships between corolla diameter (predictor) and seed production metrics (response), with warming treatment included as an interaction term. Corolla diameter was selected as a proxy for flower size to examine its relationship with seed set rate and seeds per fruit. This metric reflects the vertical dimension of floral displays, which directly corresponds to the effective contact area for pollinator recognition and visitation (Paudel et al. 2016). Corolla diameter is a widely used proxy for overall flower size. On one hand, it reflects the richness of floral resources at the single-flower level; on the other hand, flower size plays an important advertising role in attracting pollinators (Paudel et al. 2016). In previous studies on *Impatiens oxyanthera*, corolla diameter was found to be significantly affected by year (Tao et al. 2024), suggesting its sensitivity to environmental variation. Therefore, we selected this trait for further analysis.

## Results

### Flowering phenology

**Figure 2.**
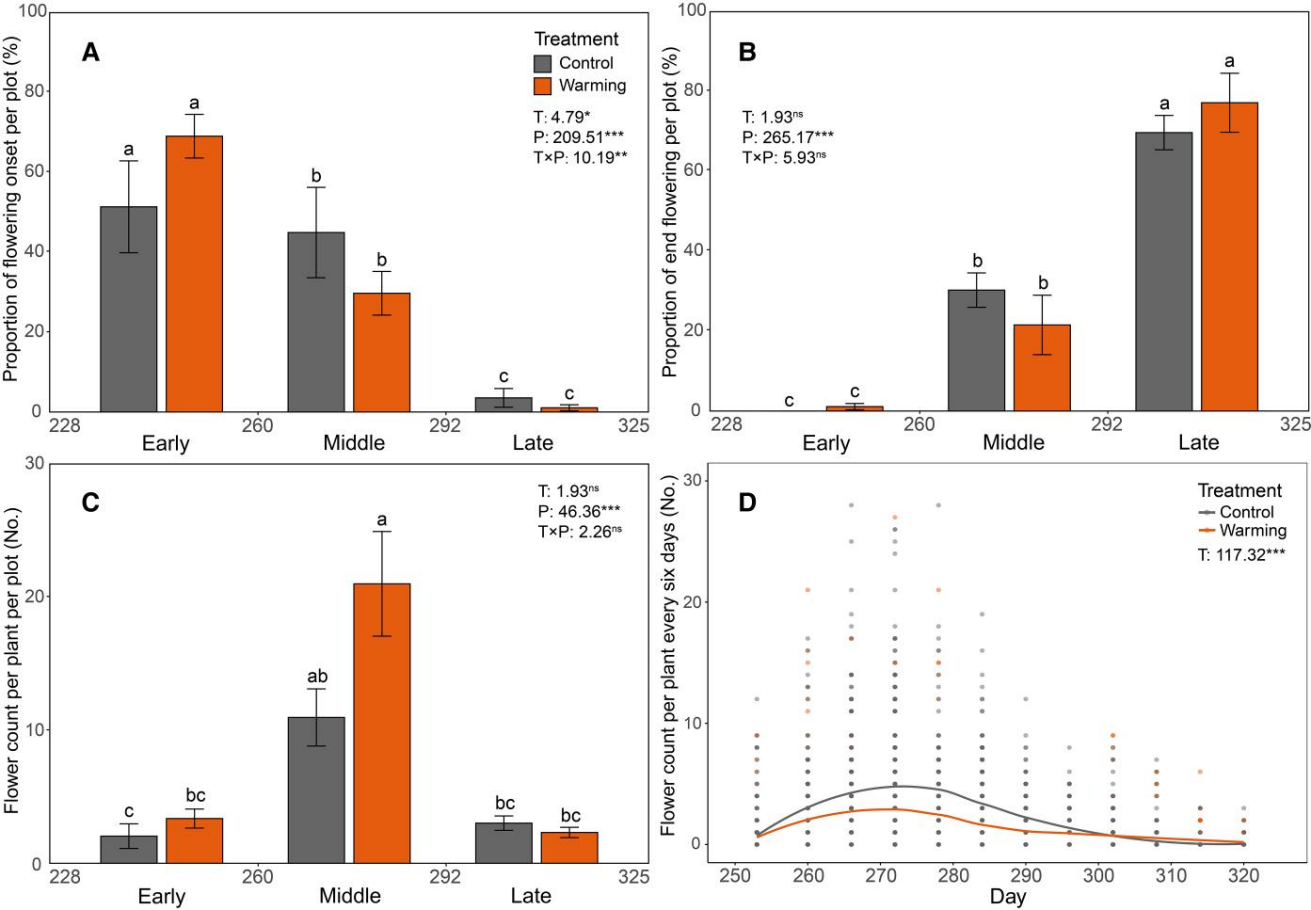
Effects of warming treatment and flowering period on flowering dynamics of *Impatiens oxyanthera*. (a) Proportion of flowering onset per plot, (b) proportion of end flowering per plot, (c) flower count per plant per plot, and (d) flower count per plant every 6 days, under control and warming treatments across early, middle, and late flowering periods.

Warming significantly delayed flowering onset time by 5.4 days and reduced the total number of flowers per plant by 35.685% but did not affect flowering end time and flowering duration (Table 1). Flowering periods significantly affect the proportion of flowering onset per plot, the proportion of end flowering per plot and the number of flowers per plant in each plot (Fig. 2a–c). The proportion of plants flowering initiation significantly decreased with advancing phenological periods regardless of warming (Fig. 2a), whereas the proportion of plants flowering termination exhibited the opposite trend (Fig. 2b). Flower number per plant in each plot during the middle period was significantly higher than that in the early and late period (Fig. 2c). The interaction between warming treatment and flowering periods significantly affected the proportion of plants flowering onset in each plot (Fig. 2a). Warming significantly reduced the number of flowers counted every 6 days during the whole flowering period by 35.708% compared with the control group (Fig. 2d).

**Table 1.** Flowering phenology and total flower number per plant under the control and warming treatments.

Index	Control	Warming	*P*-value
Flowering onset time	256.114 ± 0.784b (166)	261.680 ± 1.048a (122)	**<0**.**001**
Flowering end time	298.584 ± 0.810a (166)	300.066 ± 1.376a (122)	0.265
Flowering duration (d)	42.470 ± 1.086a (166)	38.385 ± 1.667a (122)	0.120
Total number of flowers per plant	25.770 ± 1.481a (166)	16.574 ± 1.410b (122)	**0**.**038**

Flowering phenology was calculated from 1 January 2023 as the first day. Data in the table are means ± standard error, data in parentheses represent replicates, and lowercase letters indicate significant differences between treatments in the same line. The *P* value of the statistical test is <0.05, indicating that the effect of warming was significant. Significant differences are indicated in bold.

### Flower traits

**Figure 3.**
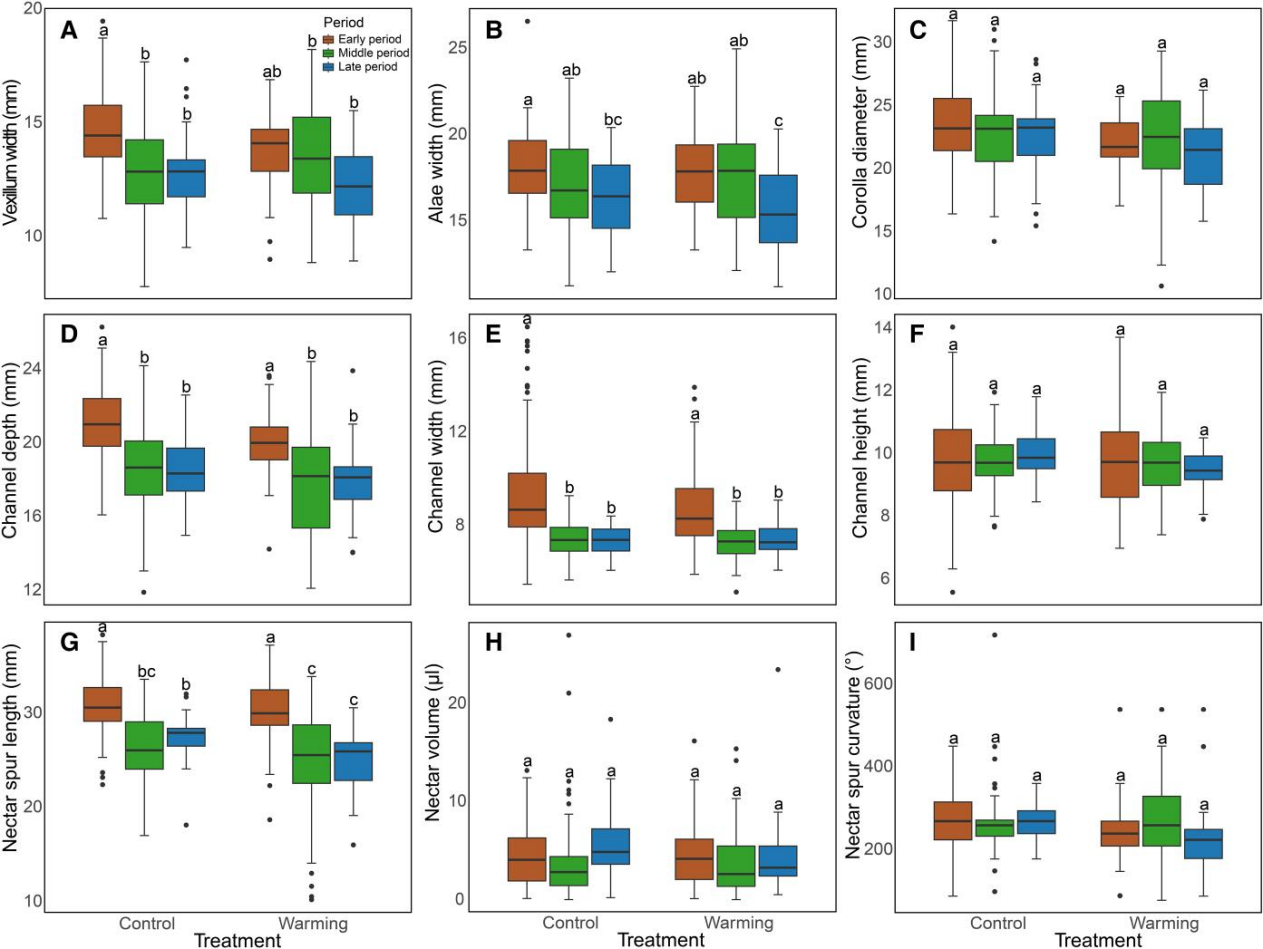
Comparisons of floral traits of *Impatiens oxyanthera* under six experimental conditions (two warming treatments × three flowering periods). Panels show (a) vexillum width, (b) alae width, (c) corolla diameter, (d) channel depth, (e) channel width, (f) channel height, (g) nectar spur length, (h) nectar volume, and (i) nectar spur curvature.

Flowering period but not warming treatment and its interaction with warming significantly affected vexillum width. Under the control conditions, the vexillum of flowers during early period was significantly 11.49% and 11.16% wider than that during middle and late periods, respectively. However, under the warming conditions, there was no significant difference in vexillum width among the three periods (Fig. 3a). Flowering period significantly affected alae width, decreasing by 12.72% and 9.19% during late period compared with that during early and middle periods (Fig. 3b). Flowers during middle and late periods had shallower and narrower channels compared with the early period (channel depth: 12.11% and 12.45% decrease, respectively; channel width: 19.93% and 19.70% decrease, respectively) (Fig. 3d and e). Warming significantly reduced nectar spur length and nectar spur curvature by 7.19% and 4.77% compared with the control, respectively (Fig. 3g and i). Nectar spur length during middle and late periods was 16.47% and 15.05% shorter than that during early period, respectively (Fig. 3g). Although there was no significant difference in nectar volume among the six experimental conditions, nectar volume was significantly 29.92% higher during late period than that during middle period (Fig. 3h, Table 2). Taken together, nectar spurs under warming became shorter and less curly, and flowers blooming at the late period became smaller.

**Table 2.** Effects of warming treatment and flowering period on flower traits of *I. oxyanthera*.

Flower traits	Warming treatment			Flowering period			Warming treatment × flowering period		
	*df*	*χ^2^*	*P*	*df*	*χ^2^*	*P*	*df*	*χ^2^*	*P*
***Flower size***									
Vexillum width	1	1.421	0.233	2	32.860	**<0**.**001**	2	10.629	**0**.**005**
Alae width	1	2.115	0.146	2	15.682	**<0**.**001**	2	3.260	0.196
Corolla diameter	1	3.773	0.052	2	2.812	0.245	2	3.750	0.153
***Pollination channel size***									
Channel depth	1	1.226	0.268	2	80.547	**<0**.**001**	2	0.115	0.944
Channel width	1	0.003	0.959	2	101.755	**<0**.**001**	2	1.198	0.550
Channel height	1	2.218	0.137	2	0.602	0.740	2	1.559	0.459
***Nectar spur shape***									
Nectar spur length	1	8.653	**0**.**003**	2	80.046	**<0**.**001**	2	5.376	0.068
Nectar spur curvature	1	4.584	**0**.**032**	2	0.014	0.993	2	4.189	0.123
Nectar volume	1	0.466	0.495	2	6.025	**0**.**049**	2	0.655	0.721

The *P* value of the statistical test was <0.05, indicating that the effect was significant. Significant effects are indicated in bold.

### Plant reproductive success

**Figure 4.**
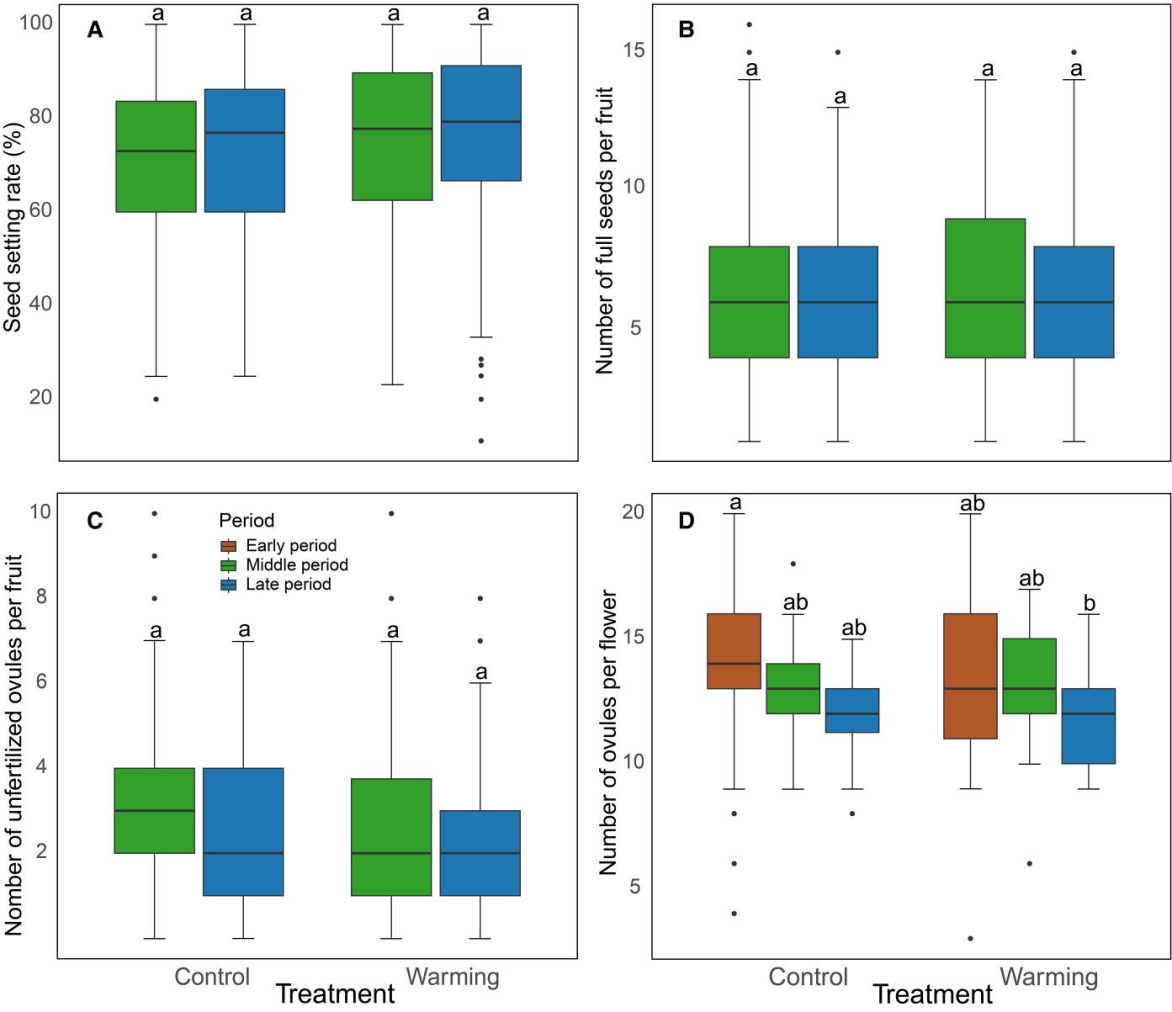
Effects of flowering period and warming treatment on reproductive success in *Impatiens oxyanthera*. (a) Seed setting rate per fruit, (b) number of filled seeds per fruit, (c) number of unfertilized ovules per fruit, and (d) number of ovules per flower are shown across three flowering periods (early, peak, and late) under two treatments (control and warming).

The warming treatment had no significant effect on the seed setting rate per fruit, number of filled seeds per fruit, number of unfertilized ovules per fruit, and number of unfertilized ovules per flower (Table 3). Flowering period significantly affected both the number of filled seeds per fruit and the number of unfertilized ovules per fruit, with decreases of 15.46% and 21.91%, respectively, during the late period compared with the middle period (Fig. 4b and c). The number of ovules per flower was significantly reduced by 14.239% during the late period compared with the early period (Fig. 4d).

**Table 3.** Effects of warming treatment and flowering period on reproductive characteristics of *I. oxyanthera*.

Reproductive characteristics	Warming treatment			Flowering period			Warming treatment × flowering period		
	*df*	*χ^2^*	*P*	*df*	*χ^2^*	*P*	*df*	*χ^2^*	*P*
Seed set per fruit	1	0.682	0.409	1	0.557	0.456	1	3.420	0.064
Filled seeds per fruit	1	0.001	0.982	1	14.296	**<0**.**001**	1	2.457	0.117
Unfertilized ovules per fruit	1	1.483	0.223	1	12.513	**<0**.**001**	1	2.564	0.109
Unfertilized ovules per flower	1	1.166	0.280	2	8.212	**0**.**016**	2	0.517	0.772

The reproductive characteristics listed in the table include seed setting rate per fruit, number of filled seeds per fruit, number of unfertilized ovules per fruit, number of unfertilized ovules per flower. The *P* value of the statistical test was <0.05, indicating that the effect was significant. Significant effects are indicated in bold.

### Correlation between flower traits

**Figure 5.**
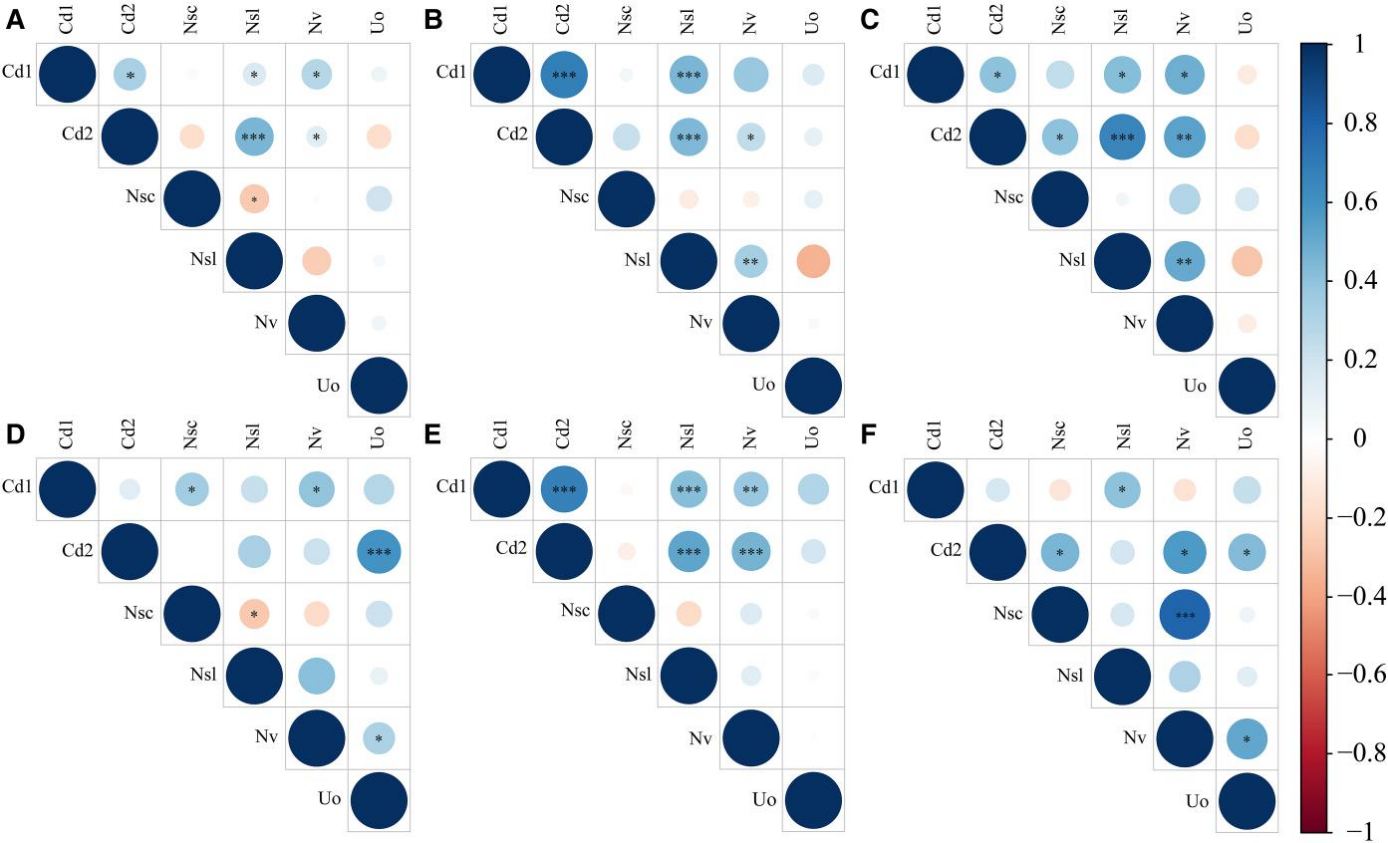
Correlation matrices of floral traits of Impatiens oxyanthera across flowering periods and warming treatments. (a–c) Show Pearson’s correlation coefficients among floral traits (Cd1: corolla diameter; Cd2: channel depth; Nsc: nectar spur curvature; Nsl: nectar spur length; Nv: nectar volume; Uo: number of ovules per flower) during the early (a), middle (b), and late (c) flowering periods in the control group. (d–f) Represent the same correlations during the early (d), middle (e), and late (f) flowering periods under the warming treatment. Blue circles indicate positive correlations and red circles indicate negative correlations.

We compared floral trait correlations across the three flowering time-periods under control and warming treatments (Fig. 5) and further assessed group differences using bootstrap correlation comparison (Supplementary Table S1). Under control conditions, significant differences in correlations were observed between the early and middle time-periods, specifically between corolla diameter and both channel depth and nectar spur curvature; as well as between nectar spur length and both nectar volume and number of ovules per flower (Fig. 5a and b). Between the early and late time periods, significant differences were found in correlations between channel depth and both nectar spur length and nectar spur curvature, and also between nectar spur length and nectar volume (Fig. 5a and c).

Under warming conditions, significant shifts in correlations were observed between channel depth and corolla diameter, nectar volume, and number of ovules per flower; as well as between nectar volume and nectar spur curvature (Fig. 5d and e). Between the middle and late time periods, the correlations between channel depth and both corolla diameter and nectar spur length differed significantly (Fig. 5e and f).

In the early time period, warming altered the correlations between corolla diameter and nectar spur curvature, channel depth and number of ovules per flower, nectar spur length and nectar volume, as well as between nectar volume and number of ovules per flower (Fig. 5a and c). In the late time period, warming significantly affected the correlations between channel depth and nectar spur length, channel depth and number of ovules per flower, and nectar volume and number of ovules per flower (Fig. 5c and f).

### Relationships between flower size and seed set

**Figure 6.**
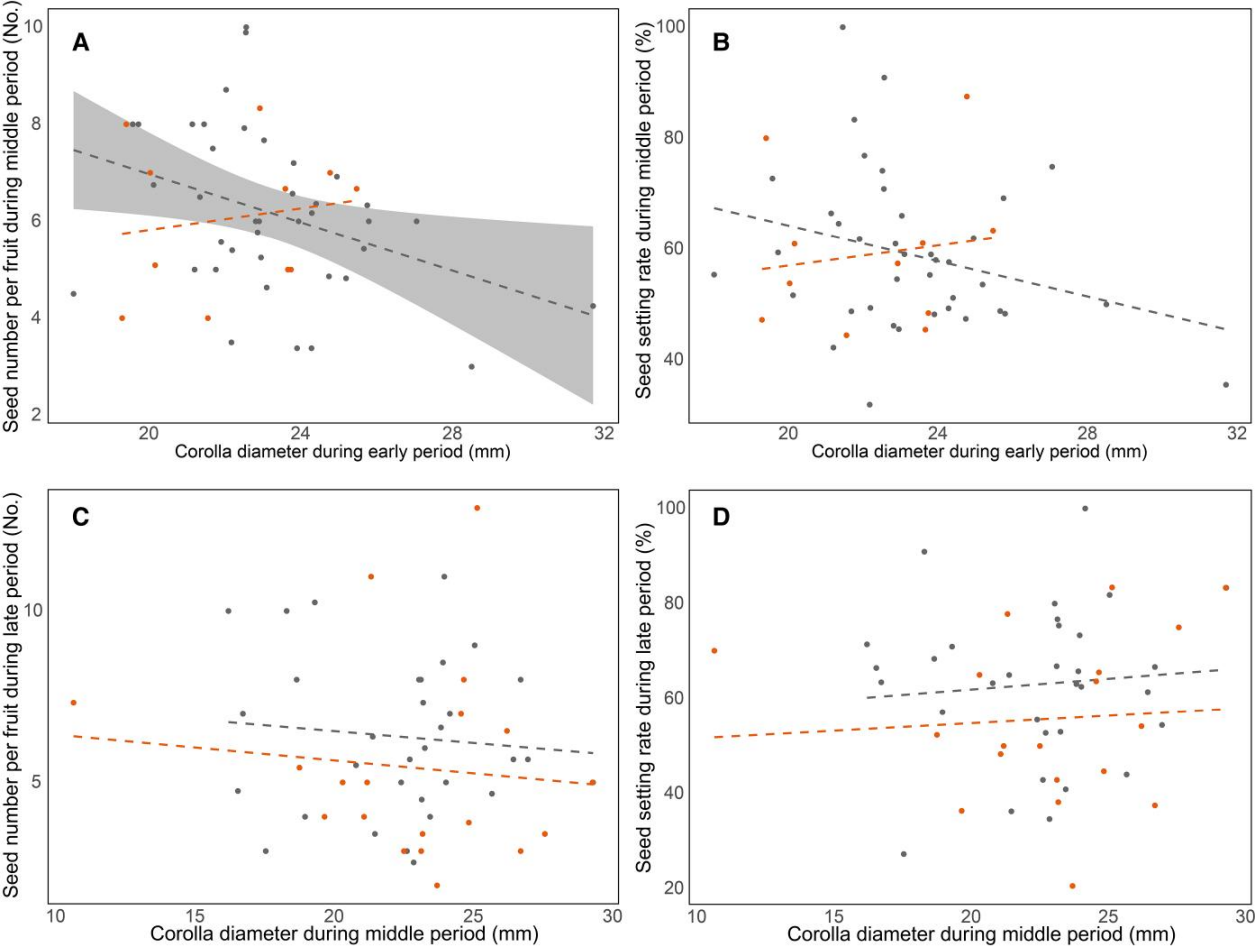
Correlations between corolla diameter and fruit reproductive output across flowering stages. (a, b) Show the correlations between corolla diameter in the early flowering period and seed number per fruit (a) and seed setting rate (b) in the middle period. (c, d) Show the correlations between corolla diameter in the middle period and seed number per fruit (c) and seed setting rate (d) in the late period. Solid lines indicate significant correlations; dashed lines indicate non-significant correlations. Orange represents the warming treatment and grey represents the control. Shaded areas denote 95% confidence intervals.

There was a significant negative correlation between corolla diameter during the early period and seed number per fruit during the middle period in the control group (*y* = 11.972 − 0.243*x*, *F* = 5.826, *P* = 0.021, Fig. 6a). However, in the warming group, there was no significant correlation between corolla diameter during early period and number of seeds per fruit during middle period (Fig. 6a). There was no significant correlation between corolla diameter during early period and seed setting rate during middle period, corolla diameter during middle period and seed number per fruit during late period, and corolla diameter during middle period and seed setting rate during late period in the control and warming groups (Fig. 6b–d).

## Discussion

Warming exerted its influence on the flowering phenology, flower number, and floral morphology of plants. In our study, warming notably postponed flowering, decreased the number of flowers per plant, and caused the nectar spurs of *I. oxyanthera* to become shorter and less curved. This could be mainly ascribed to multiple physiological stresses induced by elevated temperatures. Usually, warming advanced the flowering phenology for early-blooming species but delayed that for late-blooming species (Sherry et al. 2007). For late-blooming species, warming delayed the expression of genes related to flowering (Bianchimano et al. 2023), disrupted the equilibrium of hormones related to flowering (Castroverde and Dina 2021). Meta-analysis showed that short-term warming did not change the flower number; however, long-term warming caused a larger reduction in flower number (Zi et al. 2023), which was similar to our study results. Delayed flowering and resource shortage due to reduced net photosynthetic rate were responsible for reduction in flower number under warming (Zinn et al. 2010, Wu et al. 2011, Suzuki et al. 2014, Wang et al. 2019). Warming can also modify the morphology of the nectar spur by regulating the expression of development-related genes and reducing cell division and elongation (Mack and Davis 2015, Ballerini et al. 2020). These warming-caused changes in flowering phenology, flower number and nectar spur shape may affect the phenological matching, attraction to pollinators and mechanical fit between flowers and pollinators, thus decreasing pollination success (Miller-Rushing and Primack 2008, Hegland et al. 2009). Our study also found warming broke the normal correlation between floral features of *I. oxyanthera*, maybe because different parts of floral organs were had distinct sensitivities to temperatures (Giorno et al. 2013) or the disproportion of hormones in floral parts caused by temperature changes (Li et al. 2021*b*). This could lead to pollinators making errors in judging nectar rewards based on flower size. Our results showed no evidence that experimental warming changed the reproductive success of *I. oxyanthera* at the fruit level even if flowering phenology and nectar spur shape were significantly changed by warming. Previous studies have shown that pollinators’ flexible adaptability may play a key role, such as adjusting foraging behaviour and activity time to cope with changes in plant flowering phenology (Magrach and Montoya 2024, Basnett et al. 2025). Changes in pollinator visiting behaviours and pollination efficiency may not contribute to plant female fitness at the fruit level, because Bell (1985) found that almost all ovules of a flower were fertilized for just a visit by pollinators under the condition of fewer ovules per flower. Some plants also showed an adaptation in reproductive strategies, such as optimizing seed resource allocation or increasing pollen transfer efficiency, further improving reproductive success (Zinn et al. 2010, Wu et al. 2020). Collectively, these factors helped plants of *I. oxyanthera* maintain effective reproductive outcomes at the fruit level under warming. In addition, mild warming (+1.5°C) in our study did not reach the critical temperature point affecting reproduction, or other suitable environmental factors such as water may alleviate the negative effects of warming (Zinta et al. 2018). However, it should be noted that in our experiment, the warming treatment was not continuous due to field constraints such as unstable voltage and temperature conditions. The infrared heaters were turned off or operated intermittently for approximately 1.5 months during the growing season. This discontinuity in heating introduces a limitation to the interpretation of the warming effect, as it may have underestimated the actual physiological stress experienced by the plants or created fluctuating environmental signals that differ from constant warming scenarios. Nevertheless, such intermittent warming events may also better reflect the non-continuous nature of real-world climate warming, which often occurs in episodic patterns. We have therefore interpreted our results with caution and highlighted this limitation in both the Methods and Discussion sections.

To assess temporal variation in floral traits and reproductive performance, we divided the flowering season into three equal intervals: early, middle, and late. This standardized division allowed us to examine stage-specific plant responses to warming, considering that resource availability, pollinator activity, and environmental conditions vary throughout the season. Resource availability and environmental quality vary with flowering period, and plant reproductive characteristics and strategies also respond to these changes. In our study, *I. oxyanthera* produced larger and more abundant flowers during the early- and mid-flowering periods, potentially attracting more pollinators and thereby enhancing pollination success rates and reproductive output (Descamps et al. 2021). In contrast, during the late flowering period *I. oxyanthera* plants exhibited smaller flowers, fewer ovules, shorter nectar spurs, and higher nectar volume. Perhaps plants have completed most of their reproductive tasks, experienced physiological aging, reduced photosynthetic efficiency and resource acquisition, and faced unfavourable environmental conditions at the end of the growing season, resulting in reduced resource allocation to flowers, thus lower pollination success rates and reproductive potential (Chapin et al. 1987, Knight et al. 2005). Interestingly, flowers blooming during the late period had more nectar in our study, implying that plants attract pollinators through enhancing nectar reward. Overall, in order to adapt to reproductive pressure, plants prioritize allocating resources to organs with greater reproductive potential (Obeso 2002). We found that corolla diameter of flowers blooming during the early period was significantly negatively correlated with the number of seeds of fruits during the middle period under control conditions. In the early flowering period, larger corollas were beneficial to compensate for the lack of flowers to attract pollinators. However, if the flowers are too large, pollinators may not have close contact with the stigma and anthers, leading to a decrease in pollination efficiency (Shi et al. 2024). We also found floral trait pairs of significant correlations were more during the middle period than those during the early and late periods. This facilitates pollinators to better judge the amount of rewards through flower size and make a decision to visit during the full bloom period. Although the size and number of flowers changed with flower periods, there was no significant difference in the number of seeds and seed setting rate per fruit in *I. oxyanthera* between middle and late periods. The number of ovules per flower of *I. oxyanthera* decreasing during the later period of flowering may be responsible for this result. This phenomenon reflects an adaptation to rare pollinators, resource limitation and environmental pressures during the late flowering period. At the individual level, fruits produced from flowers blooming during the later period were difficult to mature due to adverse climate conditions at the end of the growing season. We found that the effects of warming on nectar spur length of *I. oxyanthera* were period-specific, characterized by reductions during the late flowering periods but not during the early- and mid-flowering period. During the early- and mid-flowering periods, stability in nectar spur length is maintained due to sub-threshold temperature stress and higher pollinator matching efficiency (Layek et al. 2024). In contrast, reductions observed into the late-flowering periods are driven by resource reallocation, shifts in pollinator composition, and heightened developmental sensitivity (Guddeti 2014, Tsai et al. 2018).

Our experiment compared the differences in the effects of warming on floral characteristics and plant reproductive success among the three flowering periods. The research findings suggest that the warming during the late period inflicted the most substantial damage. To gain a more comprehensive understanding of the mechanism underlying the impact of climate warming on plant reproductive success, it is essential to quantify the pollinator visitation behaviours at each flowering period. Secondly, a comparison of plant reproductive success at the individual level among different flowering periods is warranted. Finally, continuous monitoring of the long-term effects of warming on plant sexual reproduction is necessary. This will enable a more accurate assessment of the potential implications of climate change on plant reproductive processes and provide more precise evaluations of the dynamics of plant populations.

Collectively, warming has a profound impact on the flowering phenology, flower production, and nectar spur morphology and disrupts the correlations between floral traits. Compared with early- and mid-flowering periods, flowers blooming at the late period were smaller and fewer. However, plant reproductive success at the fruit level can buffer the negative effects of warming and flowering period. Moreover, plants exhibit adaptive strategies by reducing the number of ovules per flower and increasing nectar volume in the late flowering periods to optimize their reproductive opportunities.

## Supplementary Material

plaf046_Supplementary_Data

## Data Availability

Raw data and R code are available online at https://github.com/Qiao-ai406/Impatients_oxyanthera_warming_data_code/tree/efa58a0680d2d6e51bf0b758ccf4471d8c6e69d9.
