# Supplementary material for: Temporal dynamics of floral characteristics and reproduction output of Impatiens oxyanthera under warming
Source: AoB Plants. 2025 Aug 30;17(5):plaf046. doi: 10.1093/aobpla/plaf046 (PMC12448234; doi:10.1093/aobpla/plaf046)
Supplement: plaf046_Supplementary_Data [file plaf046_supplementary_data.zip › Appendix S1.pdf]

Supporting information

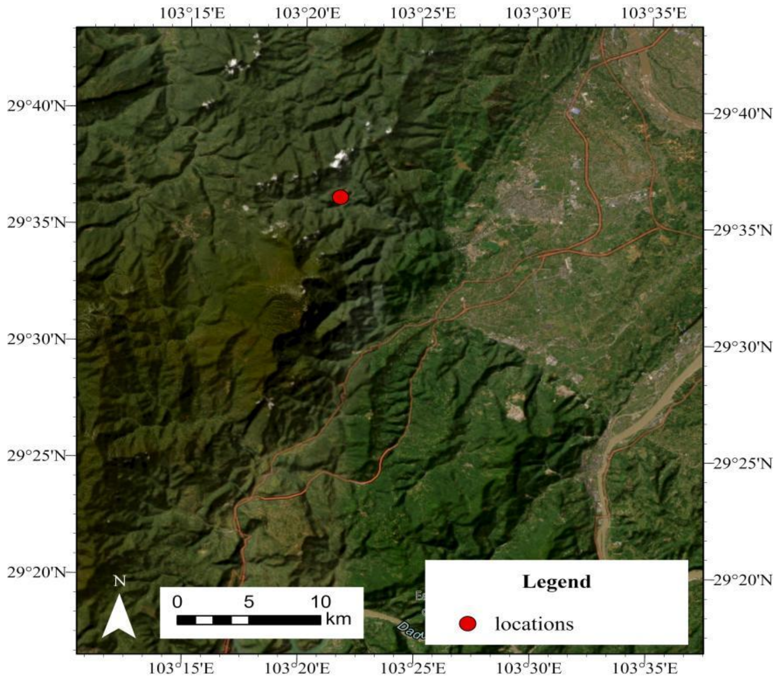

Figure S1. The red dot represents the experimental location.

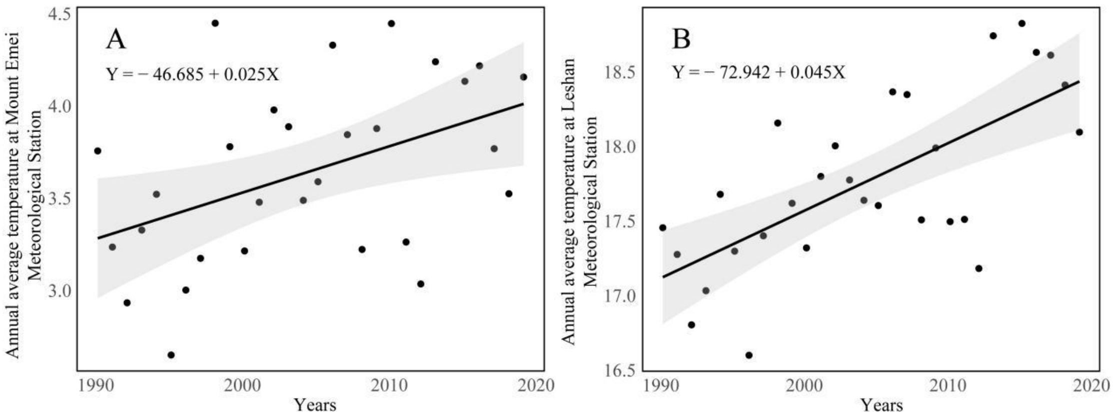

Figure S2. Panel A shows the air temperature changes over the past 30 years (1990-2019) at the meteorological station of Mount Emei (29.31° N, 103.20° E, 3047.4 m a.s.l.), and panel B displays the air temperature changes over the same period at the meteorological station of Leshan (29.34° N, 103.45° E, 404.2 m a.s.l.). The straight-line distance between these two stations is 36.8 kilometers.

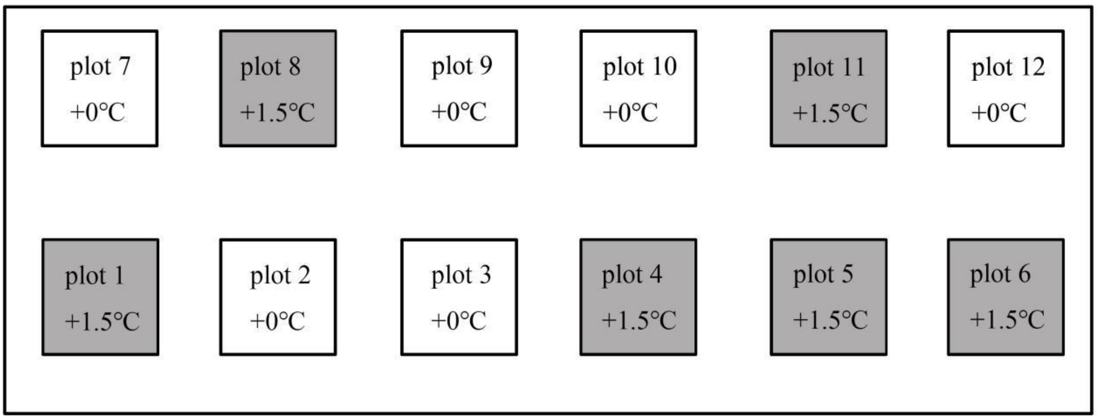

Figure S3. Temperature treatment of 12 experimental plots. Gray is a warming plot, with a 1-meter interval between each plot.

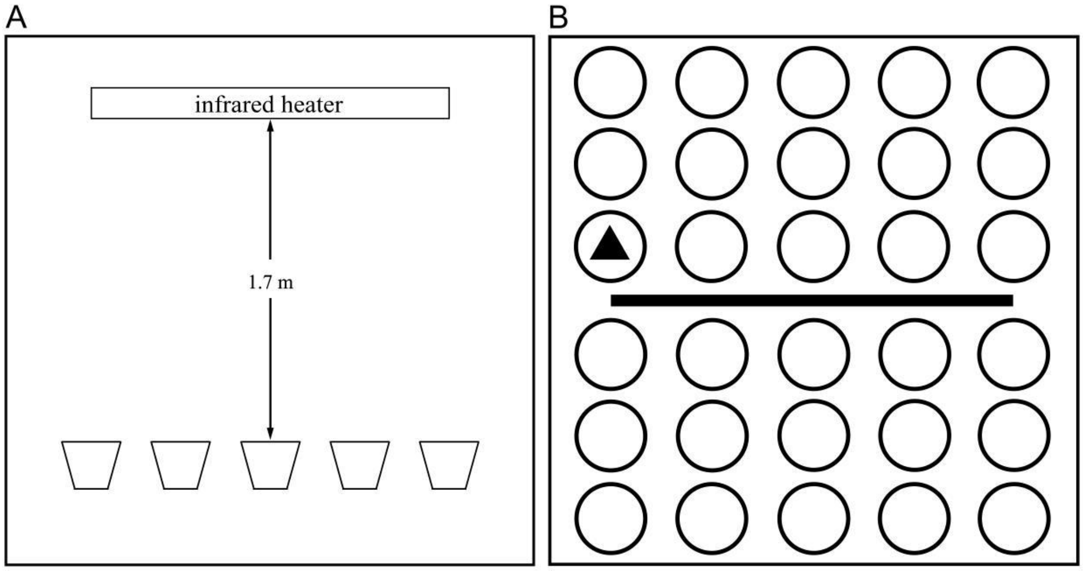

Figure S4. Schematic diagram of the experimental warming plots. Panel A illustrates a side view of the experimental plots, while Panel B displays an overhead (top-down) view. Trapezoidal shapes represent the flower pots in panel A. In panel B, hollow circles represent flower pots, the thick black line represents the projection of the infrared heater onto the plot's ground surface, and the triangle represents the location of the temperature and humidity sensors.

Table S1. The result of bootstrapped correlation comparison. Significant effects are indicated in bold.

Group1	Group2	Var1	Var2	Corr_Diff	CI_Low	CI_High	P_value
Ctrl_Early	Ctrl_Mid	Cd1	Cd2	-0.448	NA	NA	**0.002**

Ctrl_Early	Ctrl_Mid	Cd1	Nsc	-0.124	NA	NA	0.418
Ctrl_Early	Ctrl_Mid	Cd1	Nsl	-0.235	NA	NA	0.08
Ctrl_Early	Ctrl_Mid	Cd1	Nv	-0.143	NA	NA	0.258
Ctrl_Early	Ctrl_Mid	Cd1	Uo	-0.066	NA	NA	0.676
Ctrl_Early	Ctrl_Mid	Cd2	Nsc	-0.237	NA	NA	0.196
Ctrl_Early	Ctrl_Mid	Cd2	Nsl	-0.095	NA	NA	0.528
Ctrl_Early	Ctrl_Mid	Cd2	Nv	-0.073	NA	NA	0.626
Ctrl_Early	Ctrl_Mid	Cd2	Uo	-0.049	NA	NA	0.868
Ctrl_Early	Ctrl_Mid	Nsc	Nsl	-0.11	NA	NA	0.604
Ctrl_Early	Ctrl_Mid	Nsc	Nv	-0.055	NA	NA	0.624
Ctrl_Early	Ctrl_Mid	Nsc	Uo	0.09	NA	NA	0.65
Ctrl_Early	Ctrl_Mid	Nsl	Nv	-0.335	NA	NA	**0**
Ctrl_Early	Ctrl_Mid	Nsl	Uo	0.363	NA	NA	**0.046**
Ctrl_Early	Ctrl_Mid	Nv	Uo	-0.152	NA	NA	0.344
Ctrl_Mid	Ctrl_Late	Cd1	Cd2	0.264	NA	NA	0.104
Ctrl_Mid	Ctrl_Late	Cd1	Nsc	-0.191	NA	NA	0.332
Ctrl_Mid	Ctrl_Late	Cd1	Nsl	0.064	NA	NA	0.824
Ctrl_Mid	Ctrl_Late	Cd1	Nv	-0.121	NA	NA	0.488
Ctrl_Mid	Ctrl_Late	Cd1	Uo	0.24	NA	NA	0.246
Ctrl_Mid	Ctrl_Late	Cd2	Nsc	-0.185	NA	NA	0.386
Ctrl_Mid	Ctrl_Late	Cd2	Nsl	-0.221	NA	NA	0.158
Ctrl_Mid	Ctrl_Late	Cd2	Nv	-0.254	NA	NA	0.252
Ctrl_Mid	Ctrl_Late	Cd2	Uo	0.269	NA	NA	0.35
Ctrl_Mid	Ctrl_Late	Nsc	Nsl	-0.145	NA	NA	0.468
Ctrl_Mid	Ctrl_Late	Nsc	Nv	-0.347	NA	NA	0.106
Ctrl_Mid	Ctrl_Late	Nsc	Uo	-0.059	NA	NA	0.818
Ctrl_Mid	Ctrl_Late	Nsl	Nv	-0.172	NA	NA	0.212
Ctrl_Mid	Ctrl_Late	Nsl	Uo	-0.063	NA	NA	0.808
Ctrl_Mid	Ctrl_Late	Nv	Uo	0.149	NA	NA	0.458
Ctrl_Early	Ctrl_Late	Cd1	Cd2	-0.174	NA	NA	0.322
Ctrl_Early	Ctrl_Late	Cd1	Nsc	-0.312	NA	NA	0.074
Ctrl_Early	Ctrl_Late	Cd1	Nsl	-0.184	NA	NA	0.406
Ctrl_Early	Ctrl_Late	Cd1	Nv	-0.247	NA	NA	0.16
Ctrl_Early	Ctrl_Late	Cd1	Uo	0.182	NA	NA	0.268
Ctrl_Early	Ctrl_Late	Cd2	Nsc	-0.404	NA	NA	**0.034**
Ctrl_Early	Ctrl_Late	Cd2	Nsl	-0.319	NA	NA	**0.002**
Ctrl_Early	Ctrl_Late	Cd2	Nv	-0.321	NA	NA	0.114
Ctrl_Early	Ctrl_Late	Cd2	Uo	0.209	NA	NA	0.298
Ctrl_Early	Ctrl_Late	Nsc	Nsl	-0.242	NA	NA	0.132
Ctrl_Early	Ctrl_Late	Nsc	Nv	-0.405	NA	NA	0.06
Ctrl_Early	Ctrl_Late	Nsc	Uo	0.008	NA	NA	0.988
Ctrl_Early	Ctrl_Late	Nsl	Nv	-0.497	NA	NA	**0.002**
Ctrl_Early	Ctrl_Late	Nsl	Uo	0.292	NA	NA	0.15
Ctrl_Early	Ctrl_Late	Nv	Uo	0.001	NA	NA	0.99

Warm_Early	Warm_Mid	Cd1	Cd2	-0.55	NA	NA	**0**
Warm_Early	Warm_Mid	Cd1	Nsc	0.393	NA	NA	**0.02**
Warm_Early	Warm_Mid	Cd1	Nsl	-0.194	NA	NA	0.276
Warm_Early	Warm_Mid	Cd1	Nv	0.015	NA	NA	0.878
Warm_Early	Warm_Mid	Cd1	Uo	-0.005	NA	NA	0.982
Warm_Early	Warm_Mid	Cd2	Nsc	0.087	NA	NA	0.524
Warm_Early	Warm_Mid	Cd2	Nsl	-0.225	NA	NA	0.194
Warm_Early	Warm_Mid	Cd2	Nv	-0.246	NA	NA	**0.044**
Warm_Early	Warm_Mid	Cd2	Uo	0.409	NA	NA	**0.03**
Warm_Early	Warm_Mid	Nsc	Nsl	-0.067	NA	NA	0.746
Warm_Early	Warm_Mid	Nsc	Nv	-0.327	NA	NA	**0.03**
Warm_Early	Warm_Mid	Nsc	Uo	0.178	NA	NA	0.338
Warm_Early	Warm_Mid	Nsl	Nv	0.303	NA	NA	0.05
Warm_Early	Warm_Mid	Nsl	Uo	0.104	NA	NA	0.584
Warm_Early	Warm_Mid	Nv	Uo	0.283	NA	NA	0.24
Warm_Mid	Warm_Late	Cd1	Cd2	0.482	NA	NA	**0.01**
Warm_Mid	Warm_Late	Cd1	Nsc	0.077	NA	NA	0.756
Warm_Mid	Warm_Late	Cd1	Nsl	0.029	NA	NA	0.912
Warm_Mid	Warm_Late	Cd1	Nv	0.423	NA	NA	0.274
Warm_Mid	Warm_Late	Cd1	Uo	0.064	NA	NA	0.794
Warm_Mid	Warm_Late	Cd2	Nsc	-0.499	NA	NA	0.064
Warm_Mid	Warm_Late	Cd2	Nsl	0.34	NA	NA	**0.03**
Warm_Mid	Warm_Late	Cd2	Nv	0.011	NA	NA	0.818
Warm_Mid	Warm_Late	Cd2	Uo	-0.235	NA	NA	0.284
Warm_Mid	Warm_Late	Nsc	Nsl	-0.414	NA	NA	0.266
Warm_Mid	Warm_Late	Nsc	Nv	-0.431	NA	NA	0.392
Warm_Mid	Warm_Late	Nsc	Uo	-0.013	NA	NA	0.956
Warm_Mid	Warm_Late	Nsl	Nv	-0.16	NA	NA	0.376
Warm_Mid	Warm_Late	Nsl	Uo	-0.131	NA	NA	0.6
Warm_Mid	Warm_Late	Nv	Uo	-0.473	NA	NA	0.076
Warm_Early	Warm_Late	Cd1	Cd2	-0.043	NA	NA	0.852
Warm_Early	Warm_Late	Cd1	Nsc	0.466	NA	NA	0.07
Warm_Early	Warm_Late	Cd1	Nsl	-0.147	NA	NA	0.512
Warm_Early	Warm_Late	Cd1	Nv	0.464	NA	NA	0.232
Warm_Early	Warm_Late	Cd1	Uo	0.063	NA	NA	0.804
Warm_Early	Warm_Late	Cd2	Nsc	-0.384	NA	NA	0.114
Warm_Early	Warm_Late	Cd2	Nsl	0.128	NA	NA	0.538
Warm_Early	Warm_Late	Cd2	Nv	-0.253	NA	NA	0.494
Warm_Early	Warm_Late	Cd2	Uo	0.17	NA	NA	0.248
Warm_Early	Warm_Late	Nsc	Nsl	-0.468	NA	NA	0.186
Warm_Early	Warm_Late	Nsc	Nv	-0.763	NA	NA	0.06
Warm_Early	Warm_Late	Nsc	Uo	0.148	NA	NA	0.526
Warm_Early	Warm_Late	Nsl	Nv	0.152	NA	NA	0.55
Warm_Early	Warm_Late	Nsl	Uo	-0.02	NA	NA	0.894

Warm_Early	Warm_Late	Nv	Uo	-0.19	NA	NA	0.336
Ctrl_Early	Warm_Early	Cd1	Cd2	0.085	NA	NA	0.686
Ctrl_Early	Warm_Early	Cd1	Nsc	-0.441	NA	NA	**0**
Ctrl_Early	Warm_Early	Cd1	Nsl	-0.006	NA	NA	0.962
Ctrl_Early	Warm_Early	Cd1	Nv	-0.15	NA	NA	0.254
Ctrl_Early	Warm_Early	Cd1	Uo	-0.217	NA	NA	0.222
Ctrl_Early	Warm_Early	Cd2	Nsc	-0.032	NA	NA	0.736
Ctrl_Early	Warm_Early	Cd2	Nsl	0.048	NA	NA	0.828
Ctrl_Early	Warm_Early	Cd2	Nv	-0.02	NA	NA	0.86
Ctrl_Early	Warm_Early	Cd2	Uo	-0.578	NA	NA	**0**
Ctrl_Early	Warm_Early	Nsc	Nsl	0.068	NA	NA	0.664
Ctrl_Early	Warm_Early	Nsc	Nv	0.06	NA	NA	0.614
Ctrl_Early	Warm_Early	Nsc	Uo	-0.033	NA	NA	0.81
Ctrl_Early	Warm_Early	Nsl	Nv	-0.41	NA	NA	**0.006**
Ctrl_Early	Warm_Early	Nsl	Uo	-0.084	NA	NA	0.568
Ctrl_Early	Warm_Early	Nv	Uo	-0.422	NA	NA	**0.036**
Ctrl_Mid	Warm_Mid	Cd1	Cd2	0.004	NA	NA	0.976
Ctrl_Mid	Warm_Mid	Cd1	Nsc	0.074	NA	NA	0.694
Ctrl_Mid	Warm_Mid	Cd1	Nsl	0.037	NA	NA	0.776
Ctrl_Mid	Warm_Mid	Cd1	Nv	0.013	NA	NA	0.902
Ctrl_Mid	Warm_Mid	Cd1	Uo	-0.165	NA	NA	0.436
Ctrl_Mid	Warm_Mid	Cd2	Nsc	0.288	NA	NA	0.178
Ctrl_Mid	Warm_Mid	Cd2	Nsl	-0.087	NA	NA	0.534
Ctrl_Mid	Warm_Mid	Cd2	Nv	-0.205	NA	NA	0.168
Ctrl_Mid	Warm_Mid	Cd2	Uo	-0.13	NA	NA	0.666
Ctrl_Mid	Warm_Mid	Nsc	Nsl	0.11	NA	NA	0.624
Ctrl_Mid	Warm_Mid	Nsc	Nv	-0.214	NA	NA	0.142
Ctrl_Mid	Warm_Mid	Nsc	Uo	0.056	NA	NA	0.8
Ctrl_Mid	Warm_Mid	Nsl	Nv	0.209	NA	NA	0.124
Ctrl_Mid	Warm_Mid	Nsl	Uo	-0.308	NA	NA	0.164
Ctrl_Mid	Warm_Mid	Nv	Uo	0.016	NA	NA	0.928
Ctrl_Late	Warm_Late	Cd1	Cd2	0.231	NA	NA	0.282
Ctrl_Late	Warm_Late	Cd1	Nsc	0.34	NA	NA	0.232
Ctrl_Late	Warm_Late	Cd1	Nsl	0.008	NA	NA	0.984
Ctrl_Late	Warm_Late	Cd1	Nv	0.558	NA	NA	0.168
Ctrl_Late	Warm_Late	Cd1	Uo	-0.336	NA	NA	0.12
Ctrl_Late	Warm_Late	Cd2	Nsc	-0.016	NA	NA	0.892
Ctrl_Late	Warm_Late	Cd2	Nsl	0.489	NA	NA	**0.006**
Ctrl_Late	Warm_Late	Cd2	Nv	0.068	NA	NA	0.996
Ctrl_Late	Warm_Late	Cd2	Uo	-0.616	NA	NA	**0.014**
Ctrl_Late	Warm_Late	Nsc	Nsl	-0.152	NA	NA	0.712
Ctrl_Late	Warm_Late	Nsc	Nv	-0.298	NA	NA	0.538
Ctrl_Late	Warm_Late	Nsc	Uo	0.111	NA	NA	0.678
Ctrl_Late	Warm_Late	Nsl	Nv	0.224	NA	NA	0.296

Ctrl_Late	Warm_Late	Nsl	Uo	-0.381	NA	NA	0.164
Ctrl_Late	Warm_Late	Nv	Uo	-0.606	NA	NA	**0.02**

Table S2. Sample sizes (number of flowers, plots, and plants) utilized in the analyse of floral traits across experimental groups: warming treatments (Control vs. Warming) and flowering periods (Early, Mid, Late).

Traits	Control & Early	Control & Middle	Control & Late	Warming & Early	Warming & Middle	Warming & Late
Floral size	102/6/49	76/6/50	30/3/8	38/6/19	68/6/43	30/6/20
Pollination channel size	156/6/58	76/6/50	30/3/11	64/6/27	68/6/43	30/6/20
Nectar spur shape	156/6/58	76/6/50	30/3/11	64/6/27	68/6/43	30/6/20
Nectar volume	156/6/58	76/6/50	27/3/11	63/6/26	68/6/43	19/6/15

Table S3. Sample sizes (number of fruits/flowers, plots, and plants) utilized in analyses of reproductive traits across experimental groups: warming treatments (Control vs. Warming) and flowering periods (Early, Mid, Late).

Reproductive characteristics	Control & Early	Contro& Middle	Control & Late	Warming & Early	Warming & Middle	Warming & Late
Seed set per fruit	——	114/6/47	634/6/128	——	68/6/22	240/6/63
Filled seeds per fruit	——	114/6/47	634/6/128	——	68/6/22	240/6/63
Unfertilized ovules per fruit	——	114/6/47	634/6/128	——	68/6/22	240/6/63
Unfertilized ovules per flower	117/6/45	27/4/19	30/3/11	57/6/24	27/5/23	30/6/20
